# How to think about the social in psychiatric research? On language games and styles of social thought

**DOI:** 10.1007/s00127-023-02570-5

**Published:** 2023-10-14

**Authors:** Rasmus Birk, Nick Manning

**Affiliations:** 1 Department of Communication and Psychology, Aalborg University, Aalborg, Denmark; 2 Department of Global Health and Social Medicine, King’s College London, London, UK

**Keywords:** Social, Sociology, Social defeat, Language game, Epistemic justice

## Abstract

Over the last 20 years, the importance of ‘the social’ has again become a crucial theme within psychiatric research, as evidenced for example by the recent focus on the social determinants of mental health. However, what is less clear is what is meant, in this kind of research, by the very idea of the social—and what consequences those ideas have. The key purpose of the article is therefore to discuss what is often meant by the concept of ‘the social’; what different ideas of the social *do*; and what can be at stake in the different, explicit and implicit, understandings of social life that proliferate in contemporary psychiatric research. We propose that there are, roughly, three widespread styles of social thought, wherein (a) the social is seen as structural, (b) the social is seen as individual, and (c) the social is seen as relational/processual. We exemplify these by discussing examples of ‘social defeat’ and ‘therapeutic communities’, focused on what might be at stake in different understandings of social life. Lastly, we draw on the philosopher Ludwig Wittgenstein to argue that a singular understanding of ‘the social’ is not achievable.

## Introduction

How should we think about the social in psychiatric research and thought?

Around the middle of the twentieth century, the question of the social environment in the case of mental health was an important concern for American psychiatric epidemiology [1, 2]. As Allan Horwitz and Gerald Grob have argued, “The association of highly stressful environments with massive amounts of mental distress prepared the way for the social emphasis that came to dominate psychiatric epidemiology during the 1950s and 1960s.” [1, p. 693] with community studies proliferating in the following years. However, with the advent of the DSM-III in 1980, and the nosological changes it brought to psychiatry [1, 3], this emphasis on the social causes of mental health problems waned in psychiatric research in favour of approaches focusing on biological causes. There are, of course, complexities to this broad historical narrative (see for example [4–6]). But the premise for our paper is that over the last 20 years, across psychiatric and psychological research, a new emphasis on ‘the social’ and its importance for mental health has arisen, both in theoretical and empirical studies [4–6]. There seems to have been a renewed interest in the social within psychiatric thought, and within mental health research, where so-called social factors are given renewed consideration, for example as factors in the development of mental health problems [7, 8]. Of course, this trend is happening slowly and is beset with disciplinary and methodological differences [9, 10]. With this premise, the purpose of the article is to discuss what is *meant* by the notion of the social; what different ideas of the social *do*; and what is at stake in the different, explicit and implicit, understandings of social life that proliferate in contemporary research.

For example, in a much-cited paper on “The Social Determinants of Mental Health”, Jessica Allen, Reuben Balfour, Ruth Bell and Michael Marmot argue that the social determinants of (mental) health are “[…] the conditions in which people are born, live, work, and age, and the health systems they can access, which are in turn shaped by a wider set of forces: economics, social, environmental policies, and politics.” [5, p. 392]. The social determinants of mental health, in short, are the conditions of people’s lives, which again are shaped by social and societal^1^ circumstances. The social, here, begets the social and is implicated in mental health. It is not our intention here to quibble over words. Rather, we want to point to what seems like a significant lack of reflection about what, exactly, the social is.

Our concern here is mirrored in a recent article by Baptiste Brossard, Tegan Cruwys, Haochen Zhou and Gabriel Helleren-Simpson [9]. They too are interested in what is meant, concretely, when researchers write about ‘the social’ in relation to mental health. Their paper provides an analysis of 289 research articles, which linked mental health and the ‘social’. Their analysis found that the concept of the social here was used in rather uneven ways. For example, they found that the word social referred both to a person’s social position, their feelings (e.g. “social stress”), their “skills to function in everyday life”, the environment of the person (e.g. family), and many others, with the most common meaning of the social—for Brossard and colleagues—actually being “unclear” [9, p. 2].

All of this is to suggest that there is a renewed interest in the social across contemporary research on mental health problems. At the same time, it seems much less clear what the social refers to and what the consequences are of the different definitions. Does it imply the structures of society, the family, class? And, crucially, what do such definitions—be they explicit or implicit—*do?*

In the following sections, we will explore and discuss these questions. We begin by sketching out three classic ways, in social theory, of conceptualizing the social, which we then use to explore and analyse the concept of ‘social defeat’. Following this analysis, we argue that ideas of the social can act as a potential source of epistemic injustice [11] and strategic ignorance [12]. Lastly, we link this conceptual analysis to example therapeutic communities and discuss the difficulties of settling, once and for all, conceptual debates about notions such as the social.

## Three styles of thought about the social

Across the social sciences, especially sociology and ‘social theory’ there are–at a bare minimum –three styles of thought about the social. By styles of thought, we mean roughly what Ludwig Fleck called ‘thought styles’, i.e. ways of “characterizing the common features in the problem of interest to a thought collective […]” [cited in 13, p. 259]. We also want to emphasize that there are many ways of thinking about the social and many nuances of social theory which we are not able to include and elaborate on here. These three styles of thought, then, should be seen as heuristics for thinking about the social. They are as follows:

- The social as structure (Social I)
- The social as individual (Social II)
- The social as relational (or processual) (Social III)

Social I, the social as structure, stems from the sociological tradition from Marx and onwards. This tradition (broadly) sees the social (or society) as made up of structures that delimit, constrain and guide human action. Within this style of thought, the social is thus abstracted into structures, i.e. real ‘macro-level’ patterns and dynamics in the world that can be identified, and which exert real influences and pressures upon people, their employment, well-being, hopes, possibilities and so forth. This is the tradition carried on in much contemporary social epidemiology of mental health, such as work on the social determinants of mental health, which diligently has documented associations between various ‘social’ factors, such as poverty, and mental ill-health [5, 6].

The second of these notions of the social is the opposite of the first. Here, instead of seeing the social as real structures, the social is seen as made up of individual characteristics and actions (sometimes also called ‘methodological individualism’ [14]). This style of thought is visible in research which focuses on the links between individual ‘qualities’ or ‘experiences’ and how those experiences, aggregated, come to explain broader patterns. But the correlation of for example *experiences of neighbourhood safety* with mental health problems, e.g. [15], is a much different *type* of social determinant than poverty. The former centres the psychology of the individual (perception, experience) (see also [16]), while the latter centres the relations and structures in which the person is embedded.

What is shared by both Social I and Social II is that they are “[…] beholden to the idea that it is entities that come first and relations among them only subsequently […]” [17, p. 281]; that is to say, in both these styles of social thought, the focus is on reified entities (structures or individuals), rather than relations. Relations and processes, then, is what the third style of social thought focuses on. This also has a long history, from the ideas of Georg Simmel (who saw society as being constituted in the interactions between people), to the microsociological work of Erving Goffman, focused on small-scale social interactions between people, to contemporary attempts at formulating a ‘relational sociology’ [18]. A landmark (and much cited) article by Emirbayer [17] suggests that

“The very terms or units involved in a transaction derive their meaning, significance, and identity from the (changing) functional roles they play within that transaction. The latter, seen as a dynamic, unfolding process, becomes the primary unit of analysis rather than the constituent elements themselves.” [17, p. 298].

The sociologist Andrew Abbott has recently proposed a similar approach to social theory, under the name of ‘processual sociology’ [19]. For Abbott, the “[…] social world does not consist of atomic units whose interactions obey various rules, as in the thought of economists. Nor does it consist of grand social entities that shape and determine the lives of little individuals […] A processual approach begins by theorizing the making and unmaking of all these things—individuals, social entities, cultural structures, patterns of conflict—instant by instant as the social process unfolds in time. The world of processual sociology is a world of events.” [19, p. ix].

In this style of thought, we can interpret the social as an ‘emergent property’ [20], not reducible to either constituent structures or individuals. Indeed, the philosopher of biology John Dupré argues that in the case of biological systems (and, we would suggest, social systems too) ‘downward causation’ is the predominant mechanism such that the parts are themselves shaped by the whole, and not vice-versa [20]. On this relational and processual view, structuralist/individualist definitions of the social fail insofar as they project ‘real social relations’ onto external abstractions, namely structure and individual (also leading to a seemingly unsolvable problem of linking structure with the agency of individuals^2^).

These different understandings of the social come with consequences. Take, for example, poverty. Under Social I and Social II, we might understand it as material differences between groups, or as something resulting from the (rational) choices and motivations of the individual [17, p. 292]. If inequality and poverty, however, is viewed relationally, then Emirbayer argues that it should be understood as coming “largely from the solutions that elite and nonelite actors improvise in the face of recurrent organizational problems—challenges centering around control over symbolic, positional, or emotional resources.” [17, p. 292]. Thus, understanding the social as relational and processual is radical (especially so in that it challenges us to forego, e.g. variable analysis in favour of studying process and change).

While we are sympathetic towards this relational understanding, our point in this paper is less to proselytize a particular theoretical perspective, and more to suggest that there are multiple styles of social thought, each with their own implications and consequences, which may proliferate in research, excluding competing understandings. Conceptualizing the social as a reified structural pattern can show us gross societal inequalities such as poverty (but which are difficult to change). Conceptualizing the social through individualized qualities is hugely useful for its focus on agency, but simultaneously risks descending into an assemblage of various ‘exposures’ that is far from the complex ecologies of lived social life. Lastly, the process-oriented approaches of relational styles of social thought can lack the straightforwardness of other approaches, descending into endless descriptive analyses.

In the following section, we will draw on this typology to analyse an example of social thought in contemporary psychiatric research, namely the popular concept of ‘social defeat’.

## Social defeat

“Social defeat, the experience of being excluded from a majority group, is associated with increased rates of psychiatric symptoms including anxiety […] and psychotic symptoms […]. Environmental stressors such as racism, discrimination […] bullying […] and childhood adversity […] can result in the experience of social defeat. It has been proposed that the social defeat resulting from these chronic social stressors might lead to an increased risk for the development of psychotic disorders […] [24, p. 1]; references omitted

Drawn from animal studies,^3^ this concept has become increasingly popular within psychiatry over the last two decades [24], for example, [25, 26]. As the quotation exemplifies, social defeat is used to link environmental ‘stressors’ with mental health problems. Frequently proposed as an explanation for why some minority groups often have higher rates of mental health problems, e.g. [27], social defeat is frequently defined as (per the above) the ‘experience of being excluded’, as outsider status or ‘subordinate position’ [28, 29].

If we look apart from these rather imprecise definitions—it is unclear if outsider position, ‘subordinate position’ or the experience of social exclusion are the same—what should be clear here is that the social, in social defeat, closely resembles the individualistic conceptualization which we in the above termed Social II. What is present in the concept of social defeat is a completely individualized and passive understanding of life in society.

The social defeat hypothesis has been criticized thoroughly before: for being tautological [30], for being defined incoherently [29], for missing out on the complex processes by which group identity is constituted [31] and for offering only problematic and ‘thin’ understandings of the complexities of group dynamics in social settings [28]. Yet the concept persists in the face of such critiques.

To understand the endurance of this hypothesis, we will turn to the work of Linsey McGoey’s work on ‘strategic ignorance’ [12, 32] and to Miranda Fricker’s [11] work on epistemic injustices. Put somewhat shortly, McGoey’s notion of strategic ignorance explores not just what it means to know (and how those modes of knowing come about), but also the processes by which ignorance and “unknowing” are maintained (McGoey, 2012a, 2012b). Strategic ignorance, McGoey argues, helps “both to maintain and to disrupt social and political orders, allowing both governors and the governed to deny awareness of things it is not in their interest to acknowledge.” [24, p. 4].

Social defeat, in this view, is a concept that allows for a certain strategic ignorance exactly *because* it employs the individualistic understanding of sociality, e.g. “Social II”. By doing so, the concept allows researchers to think about, talk about, write about, and hypothesize about explanatory models for the differential epidemiological risk factors of particular groups, but it also allows for a particular mode of “unknowing”.

Just like the epidemiologist Nancy Krieger famously criticized epidemiology for focusing too much on webs of causation, and too little on the spiders that *spun those webs* [33], then social defeat is a concept that blinds us from seeing the ‘spider’. In research on social defeat the process of being excluded is absent—there is only a state of having been excluded, or of having obtained a ‘subordinate position’. Nor does it focus on *how* exclusion is enacted.

Only lip service is paid to the environmental stressors that frequently become mentioned—racism, poverty, bullying and so forth—in favour on an individualized focus that relies on experience. This, we will argue, is problematic insofar as it locates the problems of for example discrimination squarely in the individual’s psyche. It does not explore how, when, and where ‘social defeat’ might be something that is done to people by way of discriminatory acts and policies. Indeed, even the transformation of the notion of being excluded*,* ‘the experience of exclusion’, to the notion of being defeated, an end-state, shifts from process to passivity; allowing us to forget about the processes of exclusion. In this view, the way in which the social is operationalized and conceptualized—as structural, individual, or relational—is crucial. Styles of social thought have consequences. They proliferate, we suggest, because they can allow for strategic ignorances. In this case, shifting the onus of mental health problems back onto individuals or nearly unchangeable structures. The open question is if such risks can be mediated.

## Epistemic justice, injustice, and the therapeutic community

In this section, we suggest that, as per our analysis above, social styles of thought—especially when the social is seen as individual—risk creating strategic ignorance, but that this risk can be mediated if the social is conceptualized as relational. One approach, “therapeutic communities”, directly addresses the dangers of strategic ignorance through the systematic deployment of Social III as the core principle in thinking about mental disorder.

Strategic ignorance creates what Miranda Fricker [11] calls “epistemic injustice”, whereby the words of people living with mental disorder can be discounted by seeing them in the wrong context, such that their testimony is disbelieved or reinterpreted—a process of “testimonial injustice” common in mental health services. More generally, Fricker suggests that “hermeneutical injustice” occurs when collective interpretive resources for those utterances are lopsided, misleading or deliberately false. This can occur for example in the cases of racism and ‘white ignorance’ [34] or sexual violence [35] or indeed ‘medical ignorance’ [36]. There has also been an explosion of recent writing using these ideas of epistemic injustice amongst service users and mental health workers critical of existing services [37–39].

A relational approach, however, can provide epistemic, testimonial and hermeneutical justice through creative and liberating possibilities that Social I and II do not. How can the structural limitations and individually experienced discriminations highlighted by Social I and II, which often suffuse the experiences of people suffering from mental health problems, be not only excavated from within a definition that centres on social relations in Social III, but inform a different approach to the design of public services and support? This may seem an unlikely ideal, yet we want to argue that therapeutic communities [40] exemplify what we mean:

“Therapeutic communities (TCs) are planned social environments that see every social interaction in the life of the community as an opportunity for personal change. In keeping with a democratic approach, everyone in the community—both staff and service users—plays an active role in the therapeutic process. TCs understand that social relationships can contribute to some forms of mental distress and as such, they value the potential for relational networks to restore individual mental health. TCs mainly originated in hospital settings and have evolved into a variety of contexts: independent/voluntary communities, prisons, children’s homes and day centres, addictions and learning disabilities.” (Clarke, Manning, et al 2019).

Therapeutic communities developed in hospital settings and have evolved into a substantial number of settings in a worldwide variety of contexts as an explicitly non-medical form of mental health support and treatment. They centre on the social relations that sufferers engage in, not as a route into understanding any underlying conditions, but as the very fabric within which mental health problem exists and can be changed. Nevertheless, they originated in the 1940s with psychiatric practitioners in two different medical settings (Maxwell Jones and Tom Main), as well as with user activists in the field of addictions, and teachers in non-mainstream schools [41]. As such they have come under sustained pressure from conventional practitioners, both as to their understanding of mental disorder as primarily a disturbance of social relations, and in their disrespect of conventional professional expertise, for example encoded in evidence-based medicine through randomised controlled trials—themselves a type of strategic ignorance of the social contexts of mental disorders.

How therapeutic communities work can be best illustrated through a landmark anthropological study of Henderson Hospital, London, by Robert Rapoport in “Community as Doctor” [42]. He observed that this long-term residential facility explicitly championed a power-sharing democratic structure (with formal votes, and a patient-led ‘parliament’), close communal values for mutual care, and a high degree of behavioural permissiveness, all designed to open up and encourage social relations, which were then mercilessly discussed in open groups through a process he called ‘reality confrontation’. Maxwell Jones [43, p. 70], the hospital’s director, describes this as ‘social learning’, a ‘little understood process of change which may result from interpersonal interaction’. Thus, a tea and coffee break, lunchtime or smoking break are all considered potential therapeutic moments because of the social interactions that may occur within them. Jones famously called this “a crash course in living”.

As such these principles tried to create a version of Fricker’s “epistemic justice” whereby service users’ thoughts and words were taken seriously in terms of “testimonial justice”, and the whole community furnished the collective interpretive resources for those utterances to be understood fully through removing any unfair disadvantage when it comes to making sense of their social experiences—“hermeneutical justice” [11].

However, there are several substantial contradictions in this approach, which have played out significantly in this field, which nevertheless has continued to survive for 75 years. We highlight three of them. First, and discussed in detail in Rapoport’s book, is that members of these communities cannot remain there forever in a kind of ideal society and eventually must return to the outside world, which will see them and their problems very differently. Second, the finance and regulation of such communities require research and professional evidence, and externally qualified staff, which have contaminated and compromised their ideals within their practices, to the point of closure for some, including the original Henderson Hospital itself. Third, the very permissive design of these ‘planned environments’ have resulted in several damaging scandals over the years, which continue to cast doubt over their reputation. All these contradictions are in different ways a contest between epistemic justice and injustice. The “social distribution of epistemic resources” (i.e. who gets to define what and where) has resulted in frictions at the boundary between differing epistemologies, where staff and/or patients move in to or out of clinical settings, or where there is administrative evaluation of clinical results. Interestingly, a recent major UK government [44] report shows that this relational approach has now made very significant inroads into the criminal justice system, unencumbered by more conventional epidemiological or vernacular assumptions.

In short, then, what we want to suggest is that the styles of social thought that we engage in—in research, in theorizing, in designing therapeutic communities—matter. But this leads to a final problem, which we have skirted somewhat in the above, namely if it is possible to signify *a* meaning of the social, of finally settling this ambiguous term. Our answer to this is no—for the social is perhaps best understood as a language game.

## Concluding discussion: the social as a language game

How, then, should we think about the social? We want to end this paper by arguing, via the philosopher Ludwig Wittgenstein, that a singular, unified conceptualization of the social is impossible. What should rather be the concern, then, is increased reflexivity and precision in the style of social thought deployed.

Wittgenstein famously argued that words and concepts did not refer to an immutable, underlying reality [45]. The meaning of a word, instead, is dependent on its use, the language game it is a part of, and the rules for its use therein. For example, the meaning of the sentence “the king is vulnerable” can refer to either one’s position on a chessboard or the French monarch in the late 1700s and this depends on the language game one is part of [46, p. 21]. As Wittgenstein explains by reference to chess: “When one shows someone the king in chess and says “This is the king”, one does not thereby explain to him the use of this piece—unless he already knows the rules of the game except for this last point: the shape of the king.” [45, p. 19]. What is tricky here is that the notion of language games means that we cannot clarify our concepts in the sense of boiling them down to their essence. This, of course, goes for notions of the social too.

Drawing too on Wittgenstein, Brossard and colleagues [9] argue for the “pragmatist clarification” of the word *social*, by looking at its use, arguing that it should be seen as a “semantic array—a range of meanings and expressive connotations” [9, p. 1]. But here is the problem: if we consider the notion of the social as part of a language game whereby it receives its definition from its use, then clarification is essentially a moot point, at least if we think that clarification  *settles* matters. The forms of life wherein language is used may shift and so may the notion of the social shift with them. Conceptual (or pragmatist) clarifications can thus be useful, but they cannot get us to an essence, or final truth of a concept.

What is instead more crucial, as we have tried to show in the above, is that in all this talk of the social to be aware of, and explore, the effects of different styles of social thought: of the strategic ignorances that they may perform, of the epistemic justices and injustices they enable. In this view, our presentation of Social I, II and III is not meant to outright suggest that one is ‘truer’ than the other, but to suggest an awareness of the multiplicity of meanings that the social can take on, how such meanings can become constituted through (amongst other things) scientific activity. Finally, it suggests that these differentiated meanings of the social are useful for particular things. Seeing the social as relational, for example, seems to relate to the experiences of service users in ways that the social as individual does not. In other words, what we are arguing for is not a uniform definition of “the social”, but greater precision in how the term is deployed and greater reflexivity about what becomes, for example, ignored.

If social thought is coming alive, once again (and, of course, in many respects it was never quite gone), then researchers and practitioners in the field of mental health are faced with stark choices and dilemmas in terms of how we should understand the social. Crucially, the question of what the social is, is both a conceptual question but also a question of *stakes*. As we have hinted at in the above, there are things at stake—practically, economically, culturally, materially, politically—in the particular style of social thought which is deployed, whether it be in research, policy or activism. A closer examination of what is at stake in, for example, Social I or Social II, might reveal why particular concepts (rather than others) gain traction in institutional contexts. More broadly, there is an entire ‘social history’ of the social itself. There are many who have sought, and who still seek, to “speak in the name of society” [47, p. 88]. Modern western history is filled with not just sociologists, but doctors, psychiatrists, historians, reformists, activists, politicians, groups and others who sought to define the terrain of the society, who tried to measure the social, who spoke about the importance of community, relationships, or welfare, who developed tools and techniques to map and chart that elusive terrain of the social [47, 48]. Indeed, as Des Fitzgerald [49] has recently argued, there may be new arenas today, in which new modes of understanding the social are developing. Or, in Wittgensteinian language, new forms of life are developing and, hence, new language games for speaking about the ‘social’. These new developments—in psychiatry as elsewhere—might then be productive entry points for further explorations and creative excursions in not just invoking the social but re-making it.

## Footnotes

1 It is not particularly clear if and how the authors distinguish between the social and the societal.

2 E.g. discussed in [21, pp. 176–178]. Much of social theory discusses this dualism between structure and agency, including how to link them, e.g. [22], [23].

3 When one (large) rat physically defends its territory against a smaller rat, it is said that the smaller rat has suffered a social defeat, leading to various behavioral and physiological changes (Björkqvist, 2001).
